# Early versus late spinal decompression surgery in treatment of traumatic spinal cord injuries; a systematic review and meta-analysis

**Published:** 2017-01-11

**Authors:** Mahmoud Yousefifard, Vafa Rahimi-Movaghar, Masoud Baikpour, Parisa Ghelichkhani, Mostafa Hosseini, AliMoghadas Jafari, Heidar Aziznejad, Abbas Tafakhori

**Affiliations:** 1 Sina Trauma and Surgery Research Center, Tehran University ofMedical Sciences, Tehran, Iran.; 2 Physiology Research Center and Department of Physiology, Faculty ofMedicine, Iran University ofMedical Sciences, Tehran, Iran.; 3 Department ofMedicine, School ofMedicine, Tehran University ofMedical Sciences, Tehran, Iran.; 4 Department of Intensive Care Nursing, School of Nursing andMidwifery, Tehran University ofMedical Sciences, Tehran, Iran.; 5 Pediatric Chronic Kidney Diseases Research Center, Children’s HospitalMedical Center, Tehran University ofMedical Sciences, Tehran, Iran; 6 Department of Epidemiology and Biostatistics, School of Public Health, Tehran University ofMedical Sciences, Tehran, Iran.; 7 Department of EmergencyMedicine, School ofMedicine, Bushehr University ofMedical Sciences, Bushehr, Iran.; 8 The Persian Gulf TropicalMedicine Research Center, Bushehr University ofMedical Sciences, Bushehr, Iran.; 9 Department of Neurology, School ofMedicine, Imam Khomeini Hospital, Tehran University ofMedical Sciences, Tehran, Iran.; 10 Iranian Center of Neurological Research, Tehran University ofMedical Sciences, Tehran, Iran.

**Keywords:** Decompression, Surgical, Early Surgical Decompression, Late Surgery, Injured Spinal Cord

## Abstract

**Introduction:**

Despite the vast number of surveys, no consensus has been reached on the optimum timing of spinal decompression surgery. This systematic review and meta-analysis aimed to compare the effects of early and latespinal decompression surgery on neurologic improvement and post-surgical complications in patients with traumatic spinal cord injuries.

**Methods:**

Two independent reviewers carried out an extended search in electronic databases. Data of neurological outcome and post-surgery complication were extracted. Finally, pooled relative risk (RR) with a 95% confidence interval (CI) was reported for comparing of efficacy of early and late surgical decompression.

**Results:**

Eventually 22 studies were included. The pooled RRwas 0.77 (95% CI: 0.68-0.89)for at least one grade neurological improvement, and 0.84 (95% CI: 0.77-0.92)for at least two grade improvement. Pooled RR for surgical decompression performed within 12 hours after the injury was 0.26 (95% CI: 0.13-0.52; p<0.001), while it was 0.75 (95% CI: 0.63-0.90; p=0.002) when the procedure was performed within 24 hours, and 0.93 (95% CI: 0.76-1.14; p=0.48) when it was carried out in the first 72 hours after the injury. Surgical decompression performed within 24 hours after injury was found to be associated with significantly lower rates of post-surgical complications (RR=0.77; 95% CI: 0.68-0.86; p<0.001).

**Conclusion:**

The findings of this study indicate that early spinal decompression surgery can improve neurologic recovery and is associated with less post-surgical complications. The optimum efficacy is observed when the procedure is performed within 12 hours of the injury.

## Introduction

Spinal decompression surgery is beneficial for decreasing the probability of post spinal cord injury (SCI)neurologicalimpairments. Findings of experimental and clinical studies have confirmed thatit improves patient outcomes by preventing the activation of secondary injury mechanisms(1, 2). However, there is an ongoing controversy regarding the best time for surgical intervention. Some clinical trials are indicative of better motor and neurologic recovery with early surgical decompression compared to late interventions(2, 3), while others have shown otherwise(4), One way to reach a consensus is conducting a systematic review and meta-analysis. In this regard, two meta-analyses have been published in 2004 and 2006(5, 6).In addition, another study has been carried out in 2013 to assess different surgical schedules in SCIs, but presence of publication bias and considerable heterogeneity has kept the authors from arriving at a reliable conclusion on this matter(7).

In recent years, a significant number of clinical trials and cohort studies have compared the efficacies of early and late surgical decompression,which provide a suitable basisfor conducting a meta-analysis on human studies. In this regard, the present study aimed to compare the effects of late and early surgical decompression on motor and neurologic recovery of SCI patients through a systematic review and meta-analysis. 

## Methods

To find the maximum number of related articles, an extended search was carried out in databases of Medline (via PubMed), EMBASE (via OvidSP), CENTRAL, SCOPUS, Web of Science (BIOSIS), and ProQuestfrom January 2000 to the end of October 2015. Search strategy was based on combining terms related to “surgical decompression” with keywords related to “spinal cord injuries” (Panel 1). The keywords were selected using Mesh and EMTREE through manual search in the titles and abstracts of related articles and eventually by consulting experts. 

**Panel 1:** Keywords used for search in EMBASE and MEDLINE databases

Database	Search terms
Medline (PubMed)	("Decompression, Surgical"[Mesh] OR "Surgical Decompression*"[tiab] OR "Early versus late surgical decompression"[tiab] OR "early surgical decompression"[tiab] OR "late surgical decompression"[tiab] OR "delay* decompression"[tiab] OR "immediate decompression"[tiab] OR "Delay* treatment"[tiab] OR "Early treatment"[tiab] OR "Late surgery"[tiab] OR "Delay* surgery"[tiab]) AND (spinal cord injuries [mh] OR spinal cord injury [tiab] OR spinal cord injuries [tiab] OR spinal cord contusion [tiab] OR spinal cord transection [tiab] OR injured spinal cord [tiab] OR traumatic central cord syndrome [tiab])
EMBASE (OvidSP)	Exp "Decompression, Surgical"/ OR ("Surgical Decompression" OR "Early versus late surgical decompression" OR "early surgical decompression" OR "late surgical decompression" OR "delay decompression" OR "immediate decompression" OR "Delay treatment" OR "Early treatment" OR "Late surgery" OR "Delay surgery").ti,ab. AND exp spinal cord injuries/ OR ("spinal cord injury" OR "spinal cord injuries" OR "spinal cord contusion" OR "spinal cord transection" OR "injured spinal cord" OR "traumatic central cord syndrome").ti,ab.

In searching PubMed interface, the archived articles in PubMed Central database were also included. Other than the mentioned systematic search, manual search was performed in Google scholar and Google search engine. The authors of related articles were also contacted via email and were asked to provide us with any unpublished data, unrecorded information or unpublished dissertations they had. In cases where data were not available online, the authors were contacted. If no response was received, a reminder was sent a week later. If the author did not respond again, other authors of the article were asked for the data through social networks such as ResearchGate and LinkedIn. Bibliographies of relevant studies were also hand-searched to find further articles or unpublished data.

***Inclusion criteria:***

All the clinical trials (class I), controlled prospective cohorts (class II), case series and retrospective studies (class III) that evaluated and compared the effects of early spinal decompression surgery with late surgery on outcome of spinal cord injuries were included. Since a meta-analysis published in 2004 had evaluated the articles published before the year 2000(6), in this study only studies carried out after that were included. Sample population comprised of patients with spinal cord injuries without any gender or ethnic restrictions. Studies were included, in which the neurologic outcome was assessed based on American Spinal Injury Association (AISA) score, American Spinal Injury Association Impairment Scale (AIS), and the Frankel score. Studying patients younger than 14 years old and non-traumatic patients, not categorizing subjects into two groups of early and late interventions, using a temporal cut-off of more than 72 hours for classification of patients, and following the subjects for less than 6 months (for assessing neurological outcome) were regarded as exclusion criteria. In addition, studies that assessed post-surgical complication were included.

***Quality Assessment and Data Extraction:***

The search results were combined and duplicate studies were removed using the EndNote software (version X5, Thomson Reuters, 2011). The methodology of the studies was assessed and controlled by two independent researchers and the summaries of extracted sources were recorded in data extraction forms. In cases of disagreement, a third reviewer evaluated the findings and the inconsistency was resolved through discussion. Data collection was done blinded to the authors, journals, institutions and departments of the articles. The findings of the systematic search were recorded in a checklist designed based on the PRISMA statement guidelines (8).Study design, characteristics of sample populations (age, gender, mechanism of SCI), type of injury (complete, incomplete), etiology (motor-vehicle accidents, falling, etc.), location of injury (cervical, thoracic, lumbar), sample size, temporal cut-off point used for classification of the patients, final outcome (neurologic outcome, post-surgical complications), and possible biases were extracted. In cases of duplicate results, the study with the greater sample size was included. When the results were presented at different times, the findings of the last follow up were included. In cases that results were presented as charts, the data extraction method suggested by Sistrom and Mergo was utilized(9).

***Quality assessment***

The quality of the included studies was assessed based on the guideline proposed by the Agency for Healthcare Research and Quality’s Methods Guide for Effectiveness and Comparative Effectiveness Reviews(10). The reviewers rated the articles and classified them into three levels of good, fair, and poor based on their design, biases, sample selection, randomization, performance, and outcome report and eventually, only studies rated as fair and good were included. 

***Statistical analyses***

Data on neurologic outcome were reported in two forms in the studies. Some surveys had compared the mean and standard deviations of ASIA score or Frankel score between the two groups of early and late surgical decompression, while others had compered the improvement rate of one/two grade(s)in AIS/Frankel score between the two mentioned groups. For the studies with the first form, standardized mean differences (SMD) were calculated with a confidence interval of 95% (95% CI) based on Hedge’s g. For studies that had compared one/two grade(s) improvement in AIS/Frankel scores, data were recorded as frequency of improved or not improved patients in each group and a pooled relative risk (RR) with a confidence interval of 95% was reported.

Pooled prevalence of post-surgical complications was assessed for each group and pooled RR was calculated for comparison of early and late surgery in decreasing post-surgical complications. In order to identify publication bias, the Egger’s and Begg’s tests were used(11). Heterogeneity was assessed through I^2^ tests and a p-value of less than 0.1 along with an I^2^ greater than 50 percent were considered as positive heterogeneity. Fixed effect model was used for homogenous, and random effect model was applied for heterogeneousanalyses. Subgroup analysis was performed to recognize the source of heterogeneity. It is worth mentioning that meta-analysis was only carried out when the data were reported by at least three studies. Statistical analyses were done via STATA version 12.0 software (STATA Corporation, College Station, TX). A p value less than 0.05 was regarded as statistically significant in all the analyses. 

**Table 1:** Characteristics of included studies

Author, year	Study design	Timing (hours)	Severity	Sample size (early/late)	Age*	Sex male (%)	Location of injury	Outcome	Score	Follow up (month)
Bourassa-Moreau* et al. *2013 ^5^	RCS	24	I / C	90 / 110	47.9±17.6	77.7	C1-L2	complication	NA	Post-surgery
Bourassa-Moreau* et al. *2015 ^6^	RCS	24	C	38 / 15	43.7±18.5	91	C1-L2	Neurologic	ASIA	6
Cengiz* et al. *2008 ^7^	RCT	8	I / C	12 / 15	41.4±14.7	66.7	T8-L2	Neurologic/complication	ASIA	12
Chen* et al. *2012 ^8^	Quasi-RCT	8	I / C	99 / 110	42.1 ± 13.8	82.8	Cervical	Neurologic/complication	ASIA/AIS	12
Dobran* et al. *2015 ^12^	RCS	12	I / C	27 / 30	50.2 ± 21.3	77.2	Cervical	Neurologic	AIS	24
Dvorak* et al. *2015 ^13^	PCS	24	I / C	355 / 533	45.7	76.5	C1-L2	Neurologic/complication	ASIA	6
Ehsaei* et al. *2013 ^15^	Quasi-RCT	24	I	15 / 15	35.9±17.2	90	T11-L2	Neurologic/complication	Frankel	6
Fehlings* et al. *2012 ^18^	PCS	24	I / C	131 / 91	47.46±16.9	75.4	Cervical	Neurologic/complication	AIS	6
Guest* et al. *2002 ^21^	RCS	24	I / C	16 / 34	45 (14–77)	62	Cervical	Neurologic	ASIA	>13
Gupta* et al. *2015 ^22^	PCS	48	I / C	23 / 46	35.7±11.5	88	Cervical	Neurologic/complication	ASIA	12
Jug* et al. *2015 ^25^	PCS	8	I / C	22 / 20	48 (25.8–72.8)	81	Cervical	Neurologic	ASIA	6
Kerwin* et al. *2005 ^27^	RCS	72	I / C	174 / 125	39.6	72.6	C1-L2	complication	NA	Post-surgery
Liu* et al. *2015 ^30^	RCS	72	I / C	172 / 317	41.4±12.0	76.6	C3-C7	Neurologic/complication	Frankel	6
Lukas* et al. *2012 ^31^	RCS	24	I / C	15 / 12	NR	NR	C3-L1	Neurologic	Frankel	6
McKinley* et al. *2004 ^32^	PCS	24	I / C	33 / 140	37.65±15.83	78.8	C3-L2	Neurologic/complication	ASIA	12
Medress* et al. *2015 ^33^	RCS	72	I / C	2249 /1099	50.34	68.9	Cervical	complication	NA	Post-surgery
Pollard* et al. *2003 ^37^	RCS	24	C	86 / 242	35±15.5	NR	Thoracic	Neurologic	ASIA	12
Rahimi* et al. *2005 ^39^	RCS	24	I / C	12 / 32	26.7 ± 8.6	90	C3-L2	Neurologic	Frankel	6
Rahimi* et al. *2014 ^38^	RCT	24	I / C	15 / 18	35±12.1	71	T5-L1	Neurologic/complication	AIS	12
Sapkas* et al. *2007 ^41^	RCS	72	I / C	31 / 36	36 (16-72)	73.1	C3-C7	Neurologic	Frankel	12
Stevens* et al. *2010 ^44^	RCS	24	I / C	16 / 34	47.7±16.2	82	Cervical thoracic	Neurologic/complication	Frankel	16
Umerani* et al. *2014 ^45^	PCS	24	I / C	34 / 64	39.2 (19–65)	78.6	C3-T1	Neurologic	AIS	6

* , data were present as mean ± standard deviation or mean and (range). RCS: Retrospective cohort study; PCS: Prospective cohort study; RCT: Randomized control trial; I: Incomplete injury; C: Complete injury; NEU: Neurologic; COMP: complication; ASIA: American Spinal Injury Association; AIS: American Spinal Injury Association Impairment Scale Impairment Scale; NR: Not reported; NA: Not applicable.

## Results

***Search and screening results***

**Figure 1:**
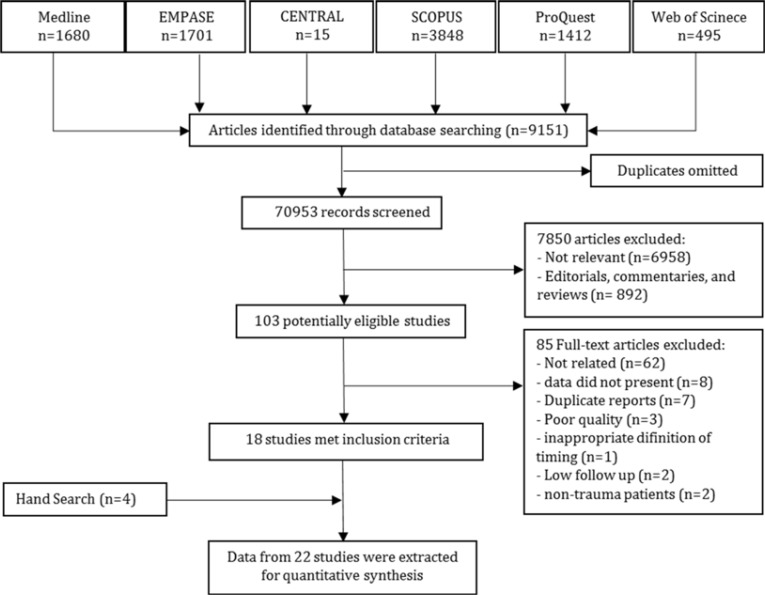
Flowchart of the study

In the extended search, 103 potentially eligible studies were screened, 29of which met the inclusion criteria. Among them, eleven studies had not presented data required for meta-analysis(12-22). Corresponding authors of these studies were contacted and three of them responded(14, 17, 21),two of which provideddata(17, 21). No answers were received from the authors of the other 8 surveys after sending two reminders. Therefore, 18 studies were included from the systematic search. Manual search yielded 4 more articles. Eventually 22 studies were included in the meta-analysis(Figure 1)(4, 17, 21, 23-41).

***Characteristics of included studies***

Included studies comprised of two randomized clinical trials (9.09%), two quasi-experimental studies (9.09%), six prospective cohorts (27.27%) and 12 retrospective cohorts (44.55%). These studies had evaluated 6803 patients (3665 subjects in the early spinal decompression surgery group and 3138 patients in the late spinal decompression surgery group). Early surgical decompression was defined as performing the operation within 8 hours in three studies (13.64%), 12 hours in one survey (4.55%), 24 hours in 13 studies (59.09%), 48 hours in one (4.55%), and 72 hours in four (18.18%). Two studies had assessed patients with complete SCIs, one had evaluated patients with incomplete SCIs (4.55%) and the rest included both types of injury (86.36%). Neurologic outcome was assessed in 9 studies (40.91%), post-surgical complications were evaluated in 3 (13.64%), and both of them were compared in 10 surveys (45.45%). Patients were followed for at least 6 months in 9 studies (40.91%), 12 months in 7 surveys (31.82%) and more than 16 months in two studies (9.09%). 19 articles were written in English , 2 in Farsi(17, 21) and one in Czech(34).Table 1 presents the characteristics of included studies. 

**Meta-analysis**

***Neurologic outcome***

Six studies had compared the neurologic score of patients between the two groups of early and late spinal decompression surgery via mean and standard deviation(26, 30, 35, 37-39),5 of which used the ASIA score(26, 30, 35, 37, 38) and one used the Frankel score(39). In this section, no publication bias was observed (p=0.99),but a moderate heterogeneity was observed (I-squared = 50.5%; p = 0.072). The pooled SMD of early and late spinal decompression surgery in neurological recovery was 0.18 (95% CI: 0.03-0.33). In other words, early surgical decompression led to moderatelybetter neurologic outcome in patients compared to late treatment.

**Figure 2:**
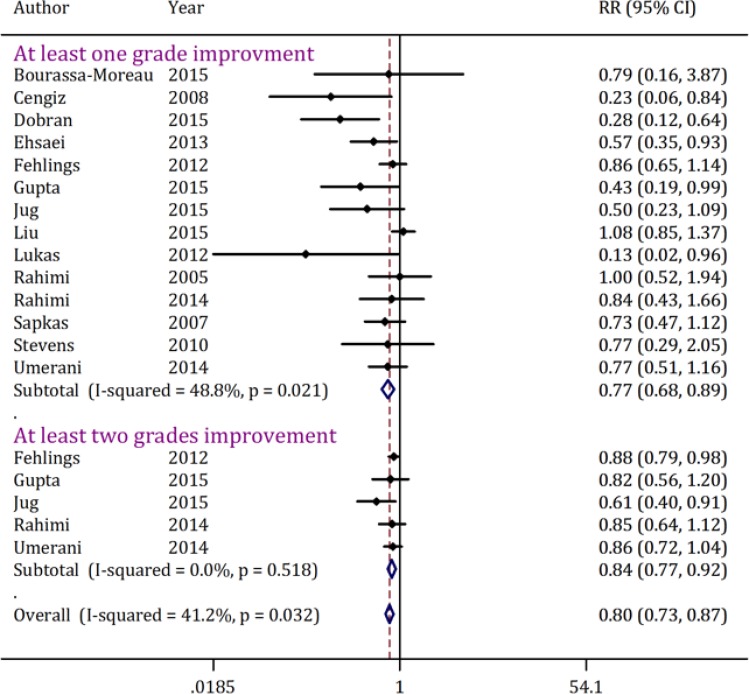
Forest plot of neuralgic improvement relative risk (RR) in individual studies and pooled estimate using the random effects model for comparing early and late surgical decompression

Neurological improvement rate was used for comparison between the two groups in 14 studies(4, 17, 21, 24, 25, 27, 29, 31, 32, 34, 38-41).The pooled RR was 0.77 (95% CI: 0.68-0.89) for at least one grade neurological improvement and 0.84 (95% CI: 0.77-0.92)for at least two grade improvement (Figure 2). No publication bias was found (p=0.66) but a moderate heterogeneity was identified (I-Squared=48.8%; p = 0.02).

Subgroup analysis was performed to find the source of heterogeneity for at least one grade improvement in neurological status (Table 2). Pooled RR yielded from clinical trials was significantly lower than that of the cohort studies (0.54 vs. 0.81). In other words,in clinical trials the efficacy reported for early spinal decompression surgery was higher than the reports of cohort studies. Pooled RR for early spinal decompression surgery in improvement of neurological outcomewas found to be 0.26 (95% CI: 0.13-0.52; p < 0.001) when the procedure was performed within 12 hours after injury, 0.75 (95% CI: 0.63-0.90; p = 0.002) when performed within 24 hours, and 0.93 (95% CI: 0.76-1.14; p = 0.48) when carried out within 72 hours. Therefore, neurologic improvement declined with the rise in the interval between injury and surgery, so that there is no significant difference between the efficacy of the treatment when performed within 72 hours or after that. Follow-up period was another effective factor. Pooled RR for studies with 6 month follow-ups was 0.87 (95% CI: 0.75-1.02; p = 0.08), while it was 0.53 (95% CI: 0.39-0.71; p < 0.001) for studies with at least 12 month follow-ups.

***Post-surgical complications***

Post-surgical complications were evaluated in 12 studies(4, 17, 23, 25, 26, 29, 31, 33, 35, 36, 38, 40). The prevalence of complications in the early spinal decompression surgery group was 0.29 (95% CI: 0.28-0.31) and in the late group was 0.38 (95% CI: 0.36-0.40). No publication bias was present (p=0.66) but a significant heterogeneity was observed (I-Squared = 65.2%; p = 0.001). Meta-analysis found the pooled RR of early spinal decompression surgery for post-surgical complications to be 0.84 (95% CI: 0.72-0.99), which indicates that the prevalence of these complications is lower in patients who had undergone early surgical decompression (p = 0.035). 

Subgroup analysis showed that the prevalence of complications reported in prospective studies was significantly lower in the early treatment group compared to the late intervention group (prevalence = 0.36 vs. 0.52; RR=0.77; p < 0.001). However, the figures reported in retrospective studies did not differ significantly between the two groups (0.28 vs. 0.34; RR=0.95; p = 0.16). Moreover, the prevalence of post-surgical complications was found to be significantly lower when the procedure was performed within 24 hours compared to later interventions (prevalence = 0.37 vs. 0.51; RR=0.77; p < 0.001). This figure was not significantly different whether the patient was treated within 72 hours of injury or after that(prevalence = 0.28 vs. 0.33; RR=0.99; p = 0.93). 

**Table 2:** Subgroup analysis of at least one improvement in neurological status for comparing early and late surgical decompression

**Characteristic**	**No of subject (early/late)**	**P for publication bias ** *	**Model**	**p for Heterogeneity (I** ^2^ **)**	**Relative risk (95% CI)**	**p**
**Overall**	553 /745	0.66	FEM	0.02 (48.8%)	0.77 (0.68-0.88)	0.02
**Data gathering method**						
Prospective	242 /269	0.55	FEM	0.22 (26.8%)	0.70 (0.68-0.89)	<0.001
Retrospective	311 /476	>0.99	REM	0.02 (59.9%)	0.85 (0.71-1.03)	0.09
**Type of study**						
Cohort	511 /697	0.92	FEM	0.03 (49.1%)	0.81 (0.70-0.93)	0.003
Control trial	42 /48	0.73	FEM	0.19 (40.1%)	0.54 (0.39-0.81)	0.003
**Time cut off** #						
8-12 hours	39 /45	>0.99	FEM	0.55 (0.0%)	0.26 (0.13-0.52)	<0.001
0-24 hours	216 /399	0.76	FEM	0.90 (0.0%)	0.75 (0.63-0.90)	0.002
0-72 hours	298 /301	0.73	REM	0.05 (66.6%)	0.93 (0.76-1.14)	0.48
**Location of injury**						
Cervical	403 /604	0.73	REM	0.02 (62.1%)	0.82 (0.71-0.94)	0.008
Thoracolumbar	42 /48	0.31	FEM	0.19 (40.1%)	0.54 (0.36-0.81)	0.003
**Follow up period**						
6 months	403 /604	0.73	REM	0.11 (40.4%)	0.87 (0.75-1.02)	0.08
≥ 12 months	42 /48	0.31	FEM	0.12 (42.2%)	0.53 (0.39-0.71)	<0.001

REM: Random effect model; FEM: Fixed effect; CI: Confidence interval.

* Based of Egger’s (Begg’s) test

# Time cut point for definition of early surgery group

**Table 3:** Subgroup analysis of post-surgery complication for comparing early and late surgical decompression

**Characteristic**	**Prevalence (95% CI)**		**Publication bias** * ** (P value)**	**Model**	**p for Heterogeneity (I** ^2^ **)**	**Relative risk (95% CI)**	**P**
	**Early group**	**Late group**					
**Overall**	0.29 (0.28-0.31)	0.38 (0.36-0.40)	0.66	FEM	0.001 (65.2%)	0.84 (0.72-0.99)	0.035
**Data gathering method**							
Prospective	0.36 (0.32-0.39)	0.52 (0.48-0.56)	0.45	FEM	0.22 (26.8%)	0.77 (0.68-0.87)	<0.001
Retrospective	0.28 (0.27-0.30)	0.34 (0.32-0.36)	0.81	REM	0.003 (75.0%)	0.95 (0.76-1.19)	0.16
**Type of study**							
Cohort	0.37 (0.30-0.45)	0.55 (0.48-0.63)	0.71	REM	0.001 (71.3%)	0.87 (0.73-1.03)	0.10
Control trial	0.29 (0.28-0.31)	0.37 (0.35-0.39)	>0.99	REM	0.05 (60.8%)	0.50 (0.21-1.19)	0.12
**Time cut off** #							
0-24 hours	0.37 (0.34-0.41)	0.51 (0.47-0.55)	0.37	FEM	0.17 (32.4%)	0.77 (0.68-0.86)	<0.001
0-72 hours	0.28 (0.26-0.29)	0.33 (0.31-0.36)	>0.99	REM	0.003 (78.6%)	0.99 (0.77-1.27)	0.93
**Location of injury**							
Cervical	0.29 (0.27-0.30)	0.36 (0.34-0.38)	>0.99	REM	0.001 (79.5%)	0.89 (0.72-1.11)	0.30
Thoracolumbar	0.11 (0.0-0.22)	0.41 (0.28-0.54)	>0.99	FEM	0.64 (0.0%)	0.33 (0.15-0.73)	0.006

REM: Random effect model; FEM: Fixed effect; CI: Confidence interval.

* , Based of Egger’s (Begg’s) test;

# Time cut point for definition of early surgery group

**Figure 3:**
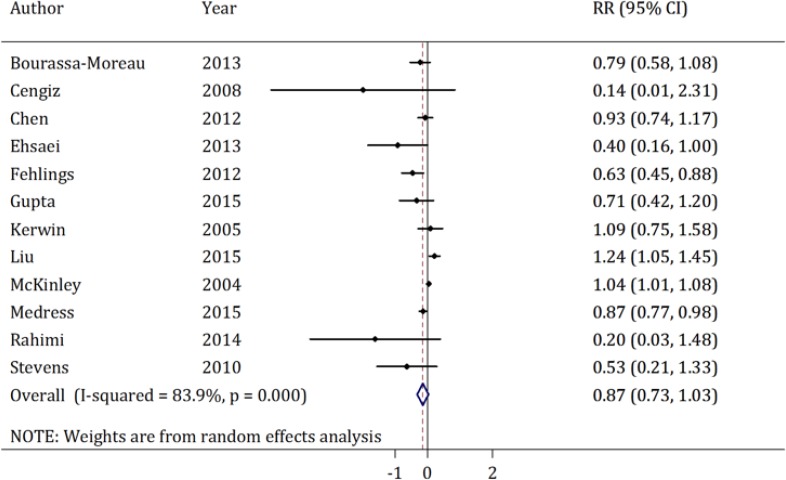
Forest plot of post-surgery complication relative risk (RR) in individual studies and pooled estimate using the random effects model for comparing early and late surgical decompression

## Discussion

In recent years,spinal decompression surgery in the early hours ofSCI has drawn major attention. Some believe that early surgical decompression in these patients can lead to better neurologic recovery and decrease post-surgical complications. However, disagreements still exist on this matter. The present meta-analysis aimed to draw a comprehensive conclusion on this subject through conducting an extended search in electronic databases. The findings of this study showed that early spinal decompression surgery,within 24 hours of injury, is associated with improved neurologic recovery and decreased post-surgical complications compared to late intervention. 

Definitions of early surgical decompression in different studies vary regarding the temporal cut-off point, which ranges from 8 to 72 hours. Accordingly, subgroup analysis was performed to assess the neurologic recovery of the patients, which indicated that longer interval between injury and spinal decompression surgery, is associated with lower treatment efficacy. Performing surgery in the first 12 hours after trauma was associated with the best neurologic recovery, while the outcomes of treatment within 72 hours and after that did not differ significantly. In this regard, it can be concluded that the optimum time for surgical decompression is the first 12 hours after injury. Considering the fact that it is not possible for most patients to undergo surgery in the first 12 hours, the cut-off point could be considered the first 24 hours.

The higher efficacy of spinal decompression surgery in the first 12 hours can be attributed to the pathologic mechanism of spinal traumatic injuries. Neural injury occurs during thefirst hours after SCI leading to hypo-perfusion, ischemia, and eventually death of neural cells (first phase of injury), while the majority of injuries occur in the second phase, which starts within few days after trauma. This phase includes apoptosis induction, formation of glial scar, central chromatolysis, disruption in expression of myelin genes, myelin destruction in remained axons, glutamate hyper-stimulation, immune cells attacking the site of lesion and release of inflammatory cytokines, endothelial injury induced by reperfusion-ischemia, and etc. (42). Hence, decompression in the first hours after injury can prevent secondary injuries or lower its severity. In line with the results of this study, van Middendrop et al. found that surgical intervention in the first 24 hours after injury is associated with better neurologic recovery, compared to the same treatment after 24 hours(7). However, the efficacy they reported was considerably higher than this study. These researchers found that surgery in the first 24 hours increases neurologic recovery by 2.5 times, while in the present meta-analysis this efficacy was found to be 1.3 times (RR=0.77). This difference could partly be attributed to the evident publication bias in the study of van Middendrop. In their meta-analysis, only two studies with a cut-off point of 24 hours were included for classification of subjects to two groups of early and late, while the present meta-analysis included 13 of such surveys. In another systematic review in 2015, Anderson et al. evaluated 9 studies aiming to assess the optimal timing of surgical decompression for acute traumatic central cord syndrome and they stated that surgery in the first 24 hours is a safe and efficient method. These authors declared that there is still not enough evidence on this matter, based on which a solid guideline could be proposed for early surgery(43).

The present meta-analysis showed that the follow-up duration can influence the yielded results. No significant difference was found between the neurologic recovery of early and late surgical decompression in studies with 6 month follow-ups (RR=0.87; 95% CI: 0.75-1.02), while evaluating the studies with at least 12 months of follow up showed significant difference between the two groups (RR=0.53; 95% CI: 0.39-0.71). This might be due to the incomplete neurologic recovery within 6 months. Although the majority of recoveries occur in the first 3 to 6 months after injury, to assess the efficacy of a treatment the maximum improvement should be considered for comparison in order to reach more reliable conclusions. Accordingly, it is suggested that the patients be followed for at least one year in the future studies. 

As presented in this meta-analysis, lower prevalence of post-surgical complications is another advantage of performing the surgery in the first 24 hours. In their overall analysis, van Middendrop et al. found the difference between the rates of post-surgical complications in early and late surgical groups to be considerable but statistically insignificant (OR=0.71; 95% CI: 0.49-1.04).(7) The overall analysis in the present study also found the mentioned difference to be near the borderline (RR=0.84; 95% CI: 0.72-0.99), but when subgroup analysis was performed for temporal cut-off point, it was illustrated that classification of patients based on a cut-off point of 72 hours can change the differences between the two groups. The differences were found to be significant when cut-off point was set to 24 hours. 

Subgroup analysis could not be performed based on severity of injury since most included studies had evaluated both complete and incomplete injuries and had not separated the two. Another limitation of this study was existence of heterogeneity between the included surveys, which led to the meta-analysis being designed based on random effect model for these cases. Although we did our best to include studies with similar methodologies and controlling for confounding factors, even in ideal situations this cannot be completely obtained. For instance, in most patients SCIs are accompanied by other injuries, a factor that can affect the final outcome of the treatments and prevalenceof post-surgical complications but is overlooked by most studies. In the present survey, only two clinical trials and two quasi-experimental studies were included and the majority of the articles were retrospective studies. Therefore, the results could be subject to selection bias. On the other hand, the retrospective nature of these studies could have influenced the collected data, which is indicative of possible bias in this section. 

Nevertheless, an extended search was conducted in electronic databases and a great effort was made to acquire data through contacting the authors, extracting information from charts and figures,and calculation of means and standard deviations. Although the last two methods are not very precise, the figures they extract are quite similar to the actual numbers,so these methodsare frequently applied in meta-analyses(44, 45). Most importantly, in addition to overall evaluation of the relation between timing of surgery and neurologic improvement, subgroup analysis was performed based on different factors, whichconsiderably helped reduce biases.

## Conclusion

The findings of this meta-analysis showed that early spinal decompression surgery is associated with better neurologic improvement and lower prevalence of post-surgical complications, compared to late intervention. The efficacy is most prominent when the surgery is performed within the first 12 hours after injury. Accordingly, it is recommended that surgical decompression be carried out in the first 12 hours after injury and postponing the procedure to later than 24 hours is associated with significant decrease in neurologic improvement and more post-surgical complications.
